# The effect of cigarette smoke exposure on vitamin D level and biochemical parameters of mothers and neonates

**DOI:** 10.1186/2251-6581-12-19

**Published:** 2013-05-11

**Authors:** Seyede Zahra Banihosseini, Azam Baheiraei, Nooshin Shirzad, Ramin Heshmat, Afshin Mohsenifar

**Affiliations:** 1 Department of Midwifery, Babol University of Medical Sciences, Babol, Iran; 2 Department of Reproductive Health, Tehran University of Medical Sciences, Tehran, Iran; 3 Endocrinology and Metabolism Research Center, Endocrinology and Metabolism Clinical Sciences Institute, Endocrinology and Metabolism Research Institute, Tehran University of Medical Sciences, Tehran, Iran; 4 TarbiatModares University, Tehran, Iran

**Keywords:** Cigarette smoke exposure, Pregnancy, 25-hydroxy vitamin D, Biochemical parameters, Cotinine

## Abstract

**Background:**

Exposure to cigarette smoke during pregnancy leads to several adverse effects on mother and child. The purpose of this study was to evaluate the effect of being a passive smoker during pregnancy on vitamin D level and related biochemical indices including parathyroid hormone, calcium, phosphorus and alkaline phosphatase in mothers and newborns.

**Methods:**

One hundred eight pregnant women and their newborns participated in a historical cohort study in two equal groups (n = 54) with and without cigarette smoke exposure. Maternal blood and urine samples and blood samples of umbilical cord were obtained in the delivery room. Concentration of 25-hydroxy vitamin D and related biochemical indices in samples of maternal and cord blood were investigated. Exposure to cigarette smoke was evaluated through questionnaire and maternal urine and umbilical cord serum cotinine levels.

**Results:**

The mean level of 25-hydroxyvitamin D in maternal serum was 9.28 ± 5.19 ng/mlin exposed and 10.75 ± 5.26 ng/ml in non-exposed group(p > 0.05). The mean concentration of 25-hydroxy vitamin D in cord serum was 10.83 ± 6.68 ng/ml in the exposed and 11.05 ± 4.99 ng/ml in the non-exposed group(p > 0.05). The exposed mothers had significantly higher parathyroid hormone level (p = 0.013), lower serum calcium (p = 0.024) and higher serum alkaline phosphatase (p = 0.024). There was a significant correlation between maternal and umbilical cord serum 25-hydroxyvitamin D within both exposed and non-exposed groups (p < 0.001).

**Conclusion:**

Maternal exposure to cigarette smoking during pregnancy negatively influences serum calcium level and increase parathyroid hormone and alkaline phosphatase in mothers.

## Background

Vitamin D is an essential nutrient required for the processes of bone metabolism and growth [1]. Need for vitamin D and calcium is higher in some periods of life including rapid growth of the embryonic period, infancy, early childhood and puberty, pregnancy, lactation and aging. Deficiency of vitamin D and calcium has more adverse effects at these times [2].

During pregnancy, major changes occur on the metabolism of vitamin D, calcium and parathyroid hormone to provide the calcium needed in fetal growth and bone mineralization especially in the last trimester of pregnancy [3,4].

Various factors such as nutrition, alcohol and caffeine consumption, the sun exposure, skin pigmentation, obesity, physical activity, clothing and seasonal variation can affect vitamin D status and bone metabolism during pregnancy [5-11]. Smoking during pregnancy has been recently focused Cigarette smoking imposes many people to environmental smoke at home, work or public places [12]. People are passive smokers in Iran at home (41.7%) and out of home (50.6%) [13]. It has been reported that 97.4% of smokers’ wives had cigarette smoke exposure during pregnancy [14]. Cigarette smoke exposure during pregnancy has effects on pregnancy outcomes such as increased spontaneous abortion [15], low birth weight [16,17], placenta detachment, premature rupture of membranes, intrauterine growth restriction [18] and vaginal bleeding [19]. Smoking during pregnancy could affect bone metabolism and leads to diminished maternal and neonatal bone mass density [20,21].

Vitamin D deficiency was seen in mothers (66.8%) and infants (93.3%) in Iran [22]. There are few studies about the impact of smoking on bone metabolism, the status of 25-hydroxy vitamin D and parathyroid hormone system during pregnancy which the majority of them have investigated the smoker women [4,20,21]. There is no study evaluating the impact of being a passive smoker during pregnancy on vitamin D and biochemical parameters in mothers and newborns.

Because of the importance of vitamin D effects on bone metabolism and fetal growth status and complications of its deficiency in pregnant women and their infants [3,5]. This study was designed to assess the effect of cigarette smoke exposure on vitamin D levels and PTH system in maternal and infant at delivery.

## Methods

Ahistorical cohort study was performed from March 2010 until May 2010. All non-smoker pregnant women who referred to a university hospital for delivery in Tehran were recruited consequently based on their reports; 54 cigarette smoke exposed subjects and 54 non-exposed subjects. The exposed group included non-smoker women who were passive smoker to a person with a regular (daily) smoking living at home or at work with them. Inclusion criteria were Iranian nationality, gestational age between 38–40 weeks and having term and healthy infant,, Exclusion criteria were history of tobacco use in mother, history of other tobacco use than cigarette in the smoker person, having chronic illnesses such as granulomatous disease, malignancies, hypertension, diabetes mellitus, hyperparathyroidism, thyroid dysfunction, kidney disease, liver or any chronic disease that affect calcium or vitamin D metabolic process, taking drugs affecting calcium or vitamin D metabolism such as phenytoin and corticosteroids during pregnancy, and recent consumption of vitamin D and calcium supplements as therapy (such as oral pills and injectable vitamin D3 and oral vitamin D2) during the past 6 months.

Demographic characteristics and data on exposure to cigarette smoke during pregnancy were recorded using cigarette smoke exposure questionnaire [23]. Subjects’ lifestyle was assessed by lifestyle questionnaire [11]. The nutrition habits and amount of calcium and vitamin D intake during pregnancy was evaluated by a questionnaire designed by the Department of Nutrition Sciences of Endocrinology and Metabolism Research Institute. Pregnancy Health Care files were used to record maternal height and weight in early pregnancy.

All mothers gave written consent to participate in the study for themselves and their infants. The study protocol was approved by ethics committee of Tehran University of Medical Sciences.

### Measurement of exposure to cigarette smoke

Cigarette exposure during pregnancy was evaluated through questionnaires [23,24] completed by researchers interviewed by subjects. They were assessed for exposure at home or work place. The number of cigarette and time of exposure were also recorded.

### Cotinine level of maternal urine and umbilical cord serum

Urine samples of mothers in hospital and umbilical cord blood of newborns were obtained in both exposed and non-exposed groups to evaluate the level of cotinine (The main metabolite of nicotine). Centrifuge to separate serum of cord blood was done in the hospital laboratory. Specimens were transferred to Toxicologic Laboratory of TarbiatModares University in cold environment and were storedat-20°C until analysis.

Cotinine levels were measured using ELISA kits (Calibotech, USA) according to the procedure set in accordance with the kit (competitive immunoassay on solid phase) and based on area under the curve. The levels were expressed according to the internal standard as a continuous quantity. Limit of Detection was 1 ng/ml. Cotinine level of urine more than 7 ng/ml was considered positive and for umbilical cord serum cut-off point was 2 ng/ml. For higher than these cut-off points regular cigarette exposures had been indicated.

### Vitamin D measurements and biochemical parameters

Fasting blood samples were collected from mothers (5 cc, at the morning of delivery day,) and umbilical cord (5 cc, after birth). Centrifuged serum samples were transferred to the Hormone Laboratory of the Endocrinology & Metabolism Research Center (EMRC) and were kept at -80°C. The serums were evaluated for 25-hydroxy vitamin D by ELISA method (IDS kit, Immunodiagnostic systems co.,UK),intra assay and inter assay coefficients of variation (CV) were 5.3% and 4.6% respectively. The normal range was considered 75–374 nmol/l (30–150 ng/ml) [25]. Vitamin D insufficiency is defined as a 25OHD concentration of 20 to 30 ng/mL (50 to 75 nmol/L),concentration of 25-hydroxy vitamin D less than 50 nmol/l (20 ng/ml) was considered deficiency and less than 25 nmol/l (10 ng/ml) was considered severe deficiency of vitamin D [25]. Parathyroid hormone also was measured by ELISA method (IDS kit, Immunodiagnostic systems co.,UK) with normal range 0.8-3.9 pmol/l with a CV of the intra assay 4.4% and inter assay 4.7%. Calcium, phosphorus, and alkaline phosphatase were measured with the auto analyzer (pars azmoon co., Iran).

### Statistical analysis

*T*-test and Mann–Whitney *U* test were used to compare quantitative measures and chi-square to compare the frequency between the two groups. The comparisons performed on two bases:A.based on maternal self report of being exposed to cigarette smoke, and B. based on cut off point selected for urine cotinine level. Indeed, there was a cross classification between the two groups applying these two approaches. The correlation between two variables was assessed using Pearson coefficient, and Spearman if needed In some cases median (quartiles) was expressed since the distributions of some parameters were not normal. All analyses were performed with SPSS v.16 software. P value less than 0.05 was considered as significant.

## Results

### Demographic data and baseline characteristics

The demographic data and baseline characteristics could be seen in Table 1 As it could be seen in Table 1 there was no significant difference between gestational age and women’s age, and also education level and jobs between exposed and non-exposed groups. Also no significant difference was found in sun exposure, exercise, caffeine consumption (tea, coffee), cola and alcohol drinks and body mass index. The consumption of dietary supplements and daily calcium and vitamin D intake during pregnancy were not significantly different between the two groups (Table 1).

**Table 1:** Demographic data and baseline characteristics

**Variable**	**Exposed (n = 54)**	**Non-exposed (n = 54)**	**p value**
Age (years)*	29.04 ± 5.69	28.07 ± 4.90	0.384
Education level			0.079
No	5(9.3%)	0	
Preliminary	8(14.8)	5(9.2)	
Guidance school	11(20.3)	11(20.3)	
High school	8(14.8)	3(5.6)	
Diploma	19(35.2)	28(51.8)	
Higher than diploma	3(5.6)	7 (13)	
Women’s job			0.113
Housewife	1(1.9)	6(11.1)	
Outdoor job	53(98.1)	48(88.9)	
BMI (kg/m^2^)			0.917
<18.5	2 (3.7%)	3 (5.6%)	
18.5-24.9	33 (60.1)	31 (57.4)	
25-29.9	14 (25.9)	16 (29.6)	
30-39.9	5 (8.3)	4 (7.4)	
Sun exposure			0.379
Every days	9 (9.3)	10 (18.5)	
Sometimes	33 (68.5)	33 (61.1)	
No exposure	12 (22.2)	11 (20.24)	
Sun exposure days in a week *	3.97 ± 1.40	4.39 ± 1.64	0.211
Exposure time in a day (minute)*	21.07 ± 8.37	19.41 ± 5.36	0.284
Exercise			0.846
Yes	23 (42.6)	24 (44.4)	
No	31 (57.4)	30 (55.6)	
Exercise days in a week *	4.18 ± 1.78	4.46 ± 1.50	0.507
Exercise time in a day (minute)*	33.06 ± 16.96	29.33 ± 12.43	0.330
Tea drinking			0.451
Yes	54 (100)	53 (98.1)	
No	0 (0)	1 (1.9)	
Consumed tea per day (cc)	465.18 ± 293.55	413.61 ± 280.30	0.353
Coffee drinking			0.728
Yes	4 (7.4)	5 (9.3)	
No	50 (92.6)	49 (90.7)	
Consumed coffee per day)cc)	2.77 ± 10.93	3.88 ± 15.31	0.665
Caffeine consumption			0.133
Yes	20 (37)	12 (22.2)	
No	34 (63)	42 (77.8)	
Caffeine consumption per day (cc)*	27.70 ± 50.10	10.46 ± 27.06	0.032
Supplement drug use			0.084
Yes	45 (83.3)	46 (85.2)	
No	9 (16.7)	8 (14.8)	
Nutritional calcium intake per day (mg/dl)*	1073.24 ± 533.40	1129**.** 22 ± 392.81	0.536

*the data are expressed as mean ± SD or median (quartile). P value less than 0.05 was considered significant.

### Exposure to cigarette smoke

All passive smoker mothers had experienced their exposure at home, not in the workplace. Husbands of the pregnant women were found to be the main source of exposure (81.4%). More than half of the exposed subjects (53.7%) had a smoker friend or relative in their home during gestation. The median number of cigarettes consumed at home was six within all trimester. The average time of exposure to cigarette smoke during pregnancy was 30 minutes per day. More than half of the positive group (59.3%) lived in one room with the smokers. More than 95% of subjects reported exposure to cigarette smoke before pregnancy.

### Maternal urine and umbilical cord serum cotinine

Geometric mean of cotinine in urine and umbilical cord serum in the exposed group was significantly higher than non-exposed (27.4 ± 29.96 ng/ml vs. 0.75 ± 2.29 and 3.71 ± 1.22 ng/ml vs. 0.404 ± 0.63, p < 0.001, respectively)

### Mothers’ Vitamin D status and biochemical parameters

We found a low level of 25(OH)vit D in both exposed and nonexposed group (according to urine cotinine level) respectively with no significant differences.(9.28 ± 5.19 ng/ml vs. 10.75 ± 5.26 ng/ml, *p* value > 0.05). Parathyroid hormone level was higher in mothers with positive cigarette exposure according to the self-reported exposure (p = 0.039) and maternal urine cotinine level (p = 0.013), (Table 2). Maternal serum calcium level was lower in the exposed group than the non-exposed subjects according to the self-reported exposure (p = 0.04) and maternal urine cotinine level (p = 0.024). There were no significant differences between groups in serum phosphorus level. Alkaline phosphatase levels in the exposed group was found to be higher than other group based on self-reported exposure (p = 0.006) and maternal urine cotinine (p = 0.024) (Table 2).

**Table 2:** Vitamin D and other biochemical factors of maternal serum (mean ± SD)

**Variable**	**Cigarette smoke exposure status**					
	**Based on maternal reports**			**Based on cut off point of urine cotinine level (ng/ml)**		
	**Exposed (n = 54)**	**Non-exposed (n = 54)**	**p value**	**Exposed (≥7) (n = 54)**	**Non-Exposed (<7) (n = 54)**	**p value**
25- hydroxy vitamin D (ng/ml)	9.31 ± 5.19	10.72 ± 5.26	0.163	9.28 ± 5.19	10.75 ± 5.26	0.149
Calcium (mg/dl)	8.71 ± 0.42	8.90 ± 0.54	0.04	8.70 ± 0.41	8.91 ± 0.54	0.024
Parathyroid (pmol/l)	1.73 ± 1.96	1.281 ± 2.72	0.039	1.78 ± 1.90	1.24 ± 2.30	0.013
Phosphorous (mg/dl)	3.54 ± 0.60	3.76 ± 0.61	0.060	3.55 ± 0.60	3.74 ± 0.610	0.106
Alkaline phosphatase (IU/l)	396.26 ± 126.84	332.48 ± 107.28	0.006	390.63 ± 124.53	338.11 ± 112.94	0.024

*the data are expressed as mean ± SD. P value less than 0.05 was considered significant.

### Infants’ Vitamin D status and biochemical parameters

Vitamin D levels in none of the infants were in normal range. The level of 25-hydroxy vitamin D in cord serum was not statistically lower in the exposed group (10.83 ± 6.68 ng/ml vs. 11.05 ± 4.99 ng/ml, *p* value > 0.05). There was no significant difference between the two groups in 25(OH)D, calcium, parathyroid hormone, phosphorus, and alkaline phosphatase levels (Table 3). There was significant correlation between 25-hydroxy vitamin D level in the maternal serum and umbilical cord serum in both exposed and non-exposed subjects (r = 0.824,P < 0.001,).

**Table 3:** Vitamin D and other biochemical factors of umbilical cord serum (mean ± SD)

Variable*	**Cigarette smoke exposure status**					
	**Based on maternal reports**			**Based oncut off point ofumblical cordcotinine level**		
	**Exposed (n = 54)**	**Non-eposed (n = 54)**	**p value**	**Exposed (≥2) (n = 55)**	**Non-exposed(<2) (n = 53)**	**p value**
25- hydroxy vitamin D (ng/ml)	10.83 ± 6.74	11.05 ± 4.94	0.847	10.83 ± 6.68	11.05 ± 4.99	0.846
Calcium (mg/dl)	10.06 ± 0.61	10.28 ± 0.86	0.134	10.04 ± 0.62	10.31 ± 0.85	0.690
Parathyroid Hormone^£^(pmol/l)	0.30(.28-.40)	0.30(.20-.43)	0.912	0.30(.30-.40)	0.30(.30-.40)	0.942
Phosphrous (mg/dl)	5.66 ± 0.72	5.71 ± 0.78	0.741	5.66 ± 0.71	5.72 ± 0.79	0.687
Alkaline phsphatase (IU/l)	390.09 ± 148.25	351.44 ± 81.74	0.097	389.67 ± 146.9	351.15 ± 82.49	0.095

*the data are expressed as mean ± SD. P value less than 0.05 was considered significant.

^£^parathyroid Hormone is expressed as median.

Vitamin D, calcium, phosphorous and alkaline phosphatase were compared by *t*-test and parathyroid by Mann–Whitney *U *test.

## Discussion

In this historical cohort study we aimed to evaluate the effects of smoke exposure during pregnancy on vitamin D levels and biochemical parameters in mothers and their neonates. The results showed that the level of 25-hydroxyvitamin D of mothers and infants were lower in exposed group, but this difference was not statistically significant. Maternal serum calcium of the exposed group was lower than the non-exposed group and the parathyroid hormone levels and alkaline phosphatase were found to be higher in the exposed group. Serum 25-hydroxyvitamin D was shown to be correlated between mothers and infants within both exposed and non-exposed groups. Diaz-Gomez et al. findings showed that smoking during pregnancy decreases serum levels of vitamin D in mothers and newborns at delivery time and also indicated that smoking reduces parathyroid hormone levels and increases serum phosphorus levels in the mothers and neonates [4]. But serum calcium and alkaline phosphatase levels of smoking mothers and their infants has been shown to have no change [4,20].

In a study on the pregnant smokers and non smokers in the Spain, the concentration of 25-hydroxyvitamin D in the maternal and umbilical cord serum was shown to be non-significantly lower in smokers [4] which was in line of our findings. Vitamin D deficiency leads to impaired intestinal calcium absorption and thus reduces the serum calcium levels [26]. The level of vitamin D in the two groups was low, and it was followed by significant reduced serum calcium levels in the exposed group. This finding could be due to direct interaction between cigarette materials including nicotine and calcium receptors which leads to impaired intestinal calcium absorption and consequently reduced calcium levels. Increased levels of parathyroid hormone and alkaline phosphatase found in the exposed group could be resulted from the effect of reduced serum calcium levels. Elevated parathyroid hormone causes increased bone turnover resulting in higher serum alkaline phosphatase levels [26]. Similar findings had been reported in a cohort study which found higher level of parathyroid hormone in the smokers than non-smokers [27].

In the results of some other studies reduced serum levels of vitamin D was associated with decreased parathyroid hormone levels and subsequent increase in serum phosphorous [4,28,29]. Nicotine receptors in the parathyroid glands which inhibited the gland was stated as a probable mechanism [4]. These findings are in contrast to the findings of the present study. These controversies could be due to vitamin D deficiency in pregnant women in the present study and low vitamin D in their diets. This deficiency was higher in the exposed group. Several studies have reported severe vitamin D deficiency in the general population [30-32] and pregnant women [22,33] in Iran. There are numerous confounding factors which are effective on vitamin D reduction [9,11,22]. In our study Vitamin D deficiency was seen in both groups so the effect of smoke on vitamin D levels could not be evaluated.

Neonatal vitamin D status is highly related to the amount of transfused vitamin D through placenta and its levels at birth [34] and maternal vitamin D deficiency causes vitamin D deficiency in infants [35]. The findings of the present study also confirm this relationship; serum levels of 25-hydroxyvitamin D in mothers and infants had a significant correlation within both exposed and non-exposed groups [5,33,34,36,37]. According to findings no significant difference was found in 25- hydroxy vitamin D, calcium, parathyroid hormone, and phosphorus and serum alkaline phosphatase levels of the umbilical cord between two groups. Gomez and colleagues reported significant difference in serum 25- hydroxyl vitamin D, parathyroid hormone and phosphorus levels of umbilical cord between groups [4]. Neonates of smoker women had lower parathyroid hormone and 25- hydroxyl vitamin D and higher phosphorus level [4]. In this study, all neonates’ cord blood had low parathyroid hormone level which could be resulted from higher calcium levels of umbilical cord than mothers. When the total calcium increases, ionized calcium levels would be also increased. Higher ionized calcium leads to more decrease in the fetal parathyroid hormone levels than the mother. As limitations, in our study, parathyroid hormone levels were evaluated using amino terminal (N-terminal) method. In this method the parathyroid level of umbilical cord could be much lower and sometimes immeasurable [38].

Low sample size and no evaluation of ionized calcium seem to affect our findings so a study on a larger sample size and broader community is needed to evaluate all confounding factors.

## Conclusions

The results showed that exposure to cigarette smoke had adverse effects on pregnant women. The serum vitamin D level was not significantly different in mothers and infants between two groups, but it was lower in the exposed group. This reduced level resulted in lower calcium level and higher serum levels of parathyroid hormone and alkaline phosphatase in mothers and infants of exposed group. This was the first research evaluated the effect of cigarette smoke on vitamin D levels and biochemical parameters on pregnant passive smokers and their fetuses. The previous studies have evaluated the effect of maternal smoking on the vitamin D levels and biochemical parameters. Considering the high prevalence of exposure to cigarette smoke during pregnancy and Vitamin D deficiency in Iran further studies with larger sample size is recommended.

Considering the vulnerability of women in this critical period of life and the effects on the infant’s health, these results suggest interventions in reducing smoke exposure during pregnancy and greater use of vitamin D and calcium supplements during pregnancy offers. In addition to pregnant women, their spouse must be aware of the potential complications associated with smoking and vitamin D deficiency in this important period of life.

## Competing interests

The authors declare that they have no competing interests.

## Authors’ contributions

SZB, AB and NS contributions to concept/design, drafting of the manuscript and critical revision of the manuscript. SZB acquisition of data. RH data analysis/interpretation. AM critical revision of the manuscript. All authors read and approved the final manuscript.

## References

[B1] Holick M High prevalence of vitamin D inadequacy and implications for health MayoClin Proc 2006 81 353 373 10.4065/81.3.353 16529140

[B2] Schoenmakers I Goldberg G Prentice A Abundant sunshine and vitamin D deficiency Br J Nutr 2008 99 1171 1173 18234141 10.1017/S0007114508898662 PMC2758994

[B3] Mulligan Megan L Felton Shaili K Riek Amy E Bernal-Mizrachi C Implications of vitamin D deficiency in pregnancy and lactation Am J ObstetGynecol 2009 201 1 9 10.1016/j.ajog.2009.05.043 PMC3540805 19846050

[B4] Diaz-Gomez N Mendoza C González-González N Barroso F Jiménez-Sosa A Domenech E Clemente I Barrios Y Barrios Y Moya M Maternal smoking and the vitamin D-parathyroid hormone system during the perinatal period Pediatr 2007 151 618 623 10.1016/j.jpeds.2007.05.003 18035141

[B5] Bowyer L Catling-Paull C Diamond T Homer C Davis G Craig M Vitamin D, PTH and calcium levels in pregnant women and their neonates ClinEndocrinol 2009 70 372 377 10.1111/j.1365-2265.2008.03316.x 18573121

[B6] Bodnar L Simhan H Powers R Frank M Cooperstein E Roberts J High prevalence of vitamin D insufficiency in black and white pregnant women residing in the northern United States and their neonates J Nutr 2007 137 452 457 10.1093/jn/137.2.447 PMC4288960 17237325

[B7] Kliegman R Maredante K Behrman R Jenson H Essentials of Pediatric 2006 5 Elsevier saunders 151 152

[B8] van der Meer I Karamali N Boeke A Lips P Middelkoop B Verhoeven I Wuister J High prevalence of vitamin D deficiency in pregnant non-Western women in The Hague, Netherlands Am J ClinNutr 2006 84 350 353 10.1093/ajcn/84.1.350 16895882

[B9] Sabour H Hossein-Nezhad A Maghbooli Z Madani F Mir E Larijani B Relationship between pregnancy outcomes and maternal vitamin D and calcium intake: A cross-sectional study Gynecol Endocrinol 2006 22 585 589 10.1080/09513590601005409 17135038

[B10] Lips P Vitamin D physiology Prog Biophys Mol Biol 2006 92 4 8 10.1016/j.pbiomolbio.2006.02.016 16563471

[B11] Hashemipour S Larijani B Adibi H Javadi E Sedaghat M Pajouhi M Soltani A Shafaei AR Hamidi Z Fard AR Hossein-Nezhad A Booya F Vitamin D deficiency and causative factors in the population of Tehran BMC Public Health 2004 4 1 6 10.1186/1471-2458-4-1 15327695 PMC517720

[B12] Chen R Tunstall-Pedoe H Tavendale R Environmental tobacco smoke and lung function in employees who never smoked: the Scottish MONICA study J Occup Environ Med 2001 58 563 568 10.1136/oem.58.9.563 PMC1740185 11511742

[B13] World Health Organization WHO report on the global tobacco epidemic, The MPOWER package,fresh and alive Retrive July 2009, from http://www.who.int/entity/tobacco/mpower/mpower_report_full_2009.pdf

[B14] ShakibaZadeh E Ahmadnia H The relationship between maternal passive smoking and birth weight and height of newborns J Zanjan University Med Sci Health Serv 2004 11 37 40 Persian

[B15] Seong MW Hwang J Moon J Ryu H Kong S Um T Park JG Lee HD Neonatal hair nicotine levels and fetal exposure to paternal smoking at home Am J Epidemiol 2008 168 1140 1144 10.1093/aje/kwn231 18801888 PMC2727244

[B16] Park E Hong Y Lee K Im M Ha E Kim Y Ha M Maternal exposure to environmental tobacco smoke, GSTM1/T1 polymorphisms and oxidative stress ReprodToxicol(Elmsford, N.Y) 2008 26 197 202 10.1016/j.reprotox.2008.08.010 18834935

[B17] Ward C Lewis S Coleman T Prevalence of maternal smoking and environmental tobacco smoke exposure during pregnancy and impact on birth weight: retrospective study using millennium cohort BMC Public Health 2007 7 81 10.1186/1471-2458-7-81 17506887 PMC1884144

[B18] Jeyabalan A Powers R Durica A Harger G Roberts J Ness R Cigarette smoke exposure and angiogenic factors in pregnancy and preeclampsia Am J Hypertens 2008 21 943 947 10.1038/ajh.2008.219 18566591 PMC2613772

[B19] Blake S Murray K El-Khorazaty M Gantz M Kiely M Best D Joseph JG El-Mohandes AA Environmental tobacco smoke avoidance among pregnant African-American nonsmokers Am J Prev Med 2009 36 225 234 10.1016/j.amepre.2008.10.012 19215848 PMC2711691

[B20] Colak O Alataş O Aydoğdu S Uslu S The effect of smoking on bone metabolism: maternal and cord blood bone marker levels Clin Biochem 2002 35 247 250 10.1016/S0009-9120(02)00301-6 12074834

[B21] Godfrey K Walker-Bone K Robinson S Taylor P Shore S Wheeler T Cooper C Neonatal bone mass: influence of parental birthweight, maternal smoking, body composition, and activity during pregnancy J Bone Miner Res 2001 16 1694 1703 2001 10.1359/jbmr.2001.16.9.1694 11547840

[B22] Maghbooli Z Hossein-Nezhad A Shafaei A Karimi F Madani F Larijani B Vitamin D status in mothers and their newborns in Iran BMC Pregnancy Childbirth 2007 7 1 6 10.1186/1471-2393-7-1 17295904 PMC1808477

[B23] Baheiraei A Kharaghani R Mohsenifar A Kazemnejad A Mota A SharifiMilani H Alikhani S Factors Associated with Secondhand Smoke Exposure in Infants Tanaffos 2010 9 43 49

[B24] Baheiraei A Banihosseini SZ Heshmat R Mota A Mohsenifar A Association of self-reported passive smoking in pregnant women with cotinine level of maternal urine and umbilical cord blood at delivery Paediatr Perinat Epidemiol 2012 26 70 76 10.1111/j.1365-3016.2011.01242.x 22150710

[B25] Holick MF Vitamin D, deficiency N Engl J Med 2007 357 266 10.1056/NEJMra070553 17634462

[B26] Fauci AS Braunwald E Kasper DL Hauser SL Longo DL Jameson JL Loscalzo J Harrison's principles of internal medicine 2008 17 New York: McGraw-Hill Professional 2376

[B27] Szulc P Garnero P Claustrat B Marchand F Duboeuf F Delmas P Increased bone resorption in moderate smokers with low body weight: the Minos study J ClinEndocrinolMetab 2002 87 666 674 10.1210/jcem.87.2.8232 11836302

[B28] Need A Kemp A Giles N Morris H Horowitz M Nordin B Relationships between intestinal calcium absorption, serum vitamin D metabolites and smoking in postmenopausal women OsteoporosInt 2002 13 83 88 10.1007/s198-002-8342-9 11883410

[B29] Jorde R Saleh F Figenschau Y Kamycheva E Haug E Sundsfjord J Serum parathyroid hormone (PTH) levels in smokers and non-smokers. The fifth Tromsø study Eur J Endocrinol 2005 152 39 45 10.1530/eje.1.01816 15762185

[B30] Rabbani A Alavian S Motlagh M Ashtiani M Ardalan G Salavati A Rabbani B Rabbani A Shams S Parvaneh N Vitamin D insufficiency among children and adolescents living in Tehran, Iran J Trop Pediatr 2009 55 189 191 10.1093/tropej/fmn078 18775944

[B31] Moradzade K Larijani B Keshtkar A Hosseinnejad A Rajabian R Nabipoor I Normal values of vitamin D and prevalence of vitamin D deficiency among Iranian population J Kurdistan University Med Sci Health Serv 2006 10 33 43 Persian

[B32] Shahla A CharehSaz S Talebi R Azad E Vitamin D deficiency in 15–40 years old females in Urmia Urmia Med J 2005 16 80 83 Persian

[B33] Kazemi A Sharifi F Jafari N Mousavinasab N High prevalence of vitamin D deficiency among pregnant women and their newborns in an Iranian population J WomensHealth 2009 18 835 839 10.1089/jwh.2008.0954 19514825

[B34] Wang J Yang F Mao M Liu D Yang H Yang S High prevalence of vitamin D and calcium deficiency among pregnant women and their newborns in Chengdu, China World J Pediatr 2010 6 265 267 10.1007/s12519-010-0224-x 20706824

[B35] Sharma S Khan N Khadri A Julies P Gnanasambandam S Saroey S Jacobs B Beski S Coren M Alexander S Vitamin D in pregnancy-time for action: a paediatric audit BJOG 2009 116 1678 1682 10.1111/j.1471-0528.2009.02285.x 19681853

[B36] Nicolaidou P Hatzistamatiou Z Papadopoulou A Kaleyias J Floropoulou E Lagona E Tsagris V Costalos C Antsaklis A Low vitamin D status in mother-newborn pairs in Greece Calcif Tissue Int 2006 78 337 342 10.1007/s00223-006-0007-5 16830197

[B37] Sachan A Gupta R Das V Agarwal A Awasthi P Bhatia V High prevalence of vitamin D deficiency among pregnant women and their newborns in northern India Am J ClinNutr 2005 81 1060 1064 10.1093/ajcn/81.5.1060 15883429

[B38] Allgrove J Adami S Manning RM Horiordan JL Cytochemical bioassay of parathyroid hormone in maternal and cord blood Arch Dis Child 1985 60 110 115 10.1136/adc.60.2.110 3977382 PMC1777137

